# Health outcomes of sarcopenia: a consensus report by the outcome working group of the Global Leadership Initiative in Sarcopenia (GLIS)

**DOI:** 10.1007/s40520-025-02995-9

**Published:** 2025-03-22

**Authors:** Charlotte Beaudart, Julian Alcazar, Ivan Aprahamian, John A. Batsis, Yosuke Yamada, Carla M. Prado, Jean-Yves Reginster, Dolores Sanchez-Rodriguez, Wee Shiong Lim, Marc Sim, Stephan von Haehling, Jean Woo, Gustavo Duque, The Global Leadership Initiative in Sarcopenia (GLIS) group

**Affiliations:** 1 Public Health Aging Research & Epidemiology (PHARE) Group, Research Unit in Clinical, Pharmacology and Toxicology (URPC), Faculty of Medicine, NAmur Research Institute for LIfe Sciences (NARILIS), University of Namur, Namur, Belgium; 2 GENUD Toledo Research Group, Faculty of Sport Sciences, University of Castilla-La Mancha, Toledo, Spain; 3 Centro de Investigación Biomédica en Red Fragilidad y Envejecimiento Saludable (CIBERFES), Instituto de Salud Carlos III, Madrid, Spain; 4 Grupo Mixto de Fragilidad y Envejecimiento Exitoso UCLM-SESCAM, Universidad de Castilla-La Mancha-Servicio de Salud de Castilla-La Mancha, IDISCAM, Toledo, Spain; 5 Division of Geriatrics, Department of Internal Medicine, Jundiaí Medical School, Jundiaí, Brazil; 6 Division of Geriatric Medicine, School of Medicine, and the Department of Nutrition, Gillings School of Global Public Health, University of North Carolina at Chapel Hill School of Medicine, Chapel Hill, NC 27599 USA; 7 Department of Medicine and Science in Sports and Exercise, Graduate School of Medicine, Tohoku University, Sendai, Miyagi 980-8575 Japan; 8 Graduate School of Biomedical Engineering, Tohoku University, Sendai, Miyagi 980-8575 Japan; 9 Department of Agricultural, Food and Nutritional Science, University of Alberta, Edmonton, AB Canada; 10 Chair for Biomarkers of Chronic Diseases, College of Science, King Saud University, Riyadh, Kingdom of Saudi Arabia; 11 Rehabilitation Research Group, Hospital del Mar Medical Research Institute, 08003 Barcelona, Spain; 12 Geriatrics Department, Brugmann University Hospital, Université Libre de Bruxelles, 1020 Brussels, Belgium; 13 Geriatrics Department, Hospital Del Mar, Hospital de L’Esperança, Centre Fòrum, Parc de Salut Mar, 08029 Barcelona, Spain; 14 Institute of Geriatrics and Active Ageing, Tan Tock Seng Hospital, Singapore, Singapore; 15 Department of Geriatric Medicine, Tan Tock Seng Hospital, Singapore, Singapore; 16 School of Medical and Health Sciences, Nutrition and Health Innovation Research Institute, Edith Cowan University, Joondalup, WA Australia; 17 Medical School, University of Western Australia, Crawley, WA Australia; 18 Department of Cardiology and Pneumology, University of Göttingen Medical Center, Göttingen, Germany; 19 German Center for Cardiovascular Research (DZHK), Partner Site Lower Saxony, Göttingen, Germany; 20 Department of Medicine and Therapeutics, The Chinese University of Hong Kong, Hong Kong, China; 21 Muscle & Geroscience Group, Research Institute of the McGill University Health Centre, BoneMontreal, QC Canada; 22 Dr. Joseph Kaufmann Chair in Geriatric Medicine, Department of Medicine, McGill University, Montreal, QC Canada

**Keywords:** GLIS, Sarcopenia, Outcomes, Falls, Fractures, Quality of life, Mortality, Hospitalization, Nursing home admission, ADL, IADL, Physical performance, Mobility, Muscle mass, Muscle strength

## Abstract

The Global Leadership Initiative in Sarcopenia (GLIS) aims to standardize the definition and diagnostic criteria for sarcopenia into one unifying, common classification. Among other actions to achieve this objective, the GLIS has organized three different working groups (WGs), with the WG on outcomes of sarcopenia focusing on reporting its health outcomes to be measured in clinical practice once a diagnosis has been established. This includes sarcopenia definitions that better predict health outcomes, the preferred tools for measuring these outcomes, and the cutoffs defining normal and abnormal values. The present article synthesizes discussions and conclusions from this WG, composed of 13 key opinion leaders from different continents worldwide. Results rely on systematic reviews, meta-analyses, and relevant cohort studies in the field. With a high level of evidence, sarcopenia is significantly associated with a reduced quality of life, a higher risk of falls and fractures and a higher risk of mortality. Sarcopenia has been moderately associated with a higher risk of reduced instrumental activities of daily living (IADL). However, the GLIS WG found only inconclusive level of evidence to support associations between sarcopenia and higher risks of hospitalization, nursing home admission, mobility impairments, and reduced basic activities of daily living (ADL). This limitation underscores the scarcity of longitudinal studies, highlighting a barrier to understanding its progression and implications over time.

## Introduction

Sarcopenia, a progressive skeletal muscle disorder characterized by the loss of muscle mass and strength, represents a significant global health burden, particularly among the aging population [1–3]. Recognized as a disease by the International Classification of Diseases (ICD-10-CM) in 2016, sarcopenia, affecting up to 10% of individuals over 60 years old, has profound implications for health systems worldwide [4–6]. The societal impacts of sarcopenia are far-reaching. Directly, it contributes to increased healthcare costs due to recurrent hospitalizations and long-term care needs [7, 8]. Indirectly, it reduces the independence and quality of life of affected individuals, with patients experiencing diminished physical performance, increased risk of falls and fractures, and challenges in performing basic and instrumental activities of daily living [9–11]. Given the increasing global life expectancy and the consequent growth of the older population, sarcopenia has been identified as a public health priority requiring immediate attention [12, 13].

Interventions to prevent or treat sarcopenia typically focus on a combination of nutritional, exercise, and, more recently, pharmacological strategies [14–16]. However, despite its recognized importance, despite its recognized importance, identifying and treating individuals with sarcopenia remains an ongoing challenge as a result of decades-long differences and debates in the lack of universally accepted operational definitions. The prevalence of sarcopenia varies widely depending on the operational definition used, leading to significant differences in the individuals identified as having the condition [17]. Therefore, the effectiveness of interventions in increasing muscle mass or muscle strength is heterogeneous. Moreover, the effectiveness of these approaches in improving patient-centered outcomes, such as quality of life, remains limited [18]. This gap highlights the critical need for standardized methodologies and comprehensive research frameworks. In this context, the Global Leadership Initiative in Sarcopenia (GLIS) was established. The initiative aims to standardize the definition and diagnostic criteria for sarcopenia into one unifying common classification that would be used as the gold standard in sarcopenia assessment, promoting, therefore, high-quality research and facilitating the translation of scientific findings into clinical practice [19, 20].

To address the multifaceted challenges of sarcopenia and develop an evidence-based operational definition of sarcopenia, the GLIS has organized three different working groups (WGs) reassembling global key opinion leaders, focusing on distinct but interrelated aspects of the disease. Presently, the GLIS initiative has instituted the following WGs: (1) muscle strength, (2) muscle mass, and (3) outcomes of sarcopenia. This specific report outlines the evidence linking the GLIS definition of sarcopenia to the outcomes mentioned in GLIS, what are the measurements used for these outcomes, as well as the clinically meaningful change for these measurements, and the cutoffs defining normal and abnormal values. A collaborating project was developed, involving different experts working on these aspects. The present article synthesizes the discussions and conclusions from this WG, providing a comprehensive overview of sarcopenia’s clinical and societal implications.

## Methodology

### Working group organization and focus

First, a literature review was conducted by the three leaders of the WG outcomes of sarcopenia (C.B., G.D. and J.W.) to identify the key outcomes of sarcopenia. A list of seven outcomes was further established, reviewed and approved by the members of the GLIS. Seven sub-WGs were developed to investigate (i) the influence of sarcopenia on physical performance and mobility, (ii) the influence of sarcopenia on instrumental and basic activities of daily living, reflecting functional independence, (iii) the influence of sarcopenia on quality of life, (iv) the relationship between sarcopenia and fall-related fractures, (v) the prevalence and implications of sarcopenia in hospitalized populations, (vi) the role of sarcopenia in predicting nursing home admissions and (vii) the association between sarcopenia and mortality,

The composition of each sub-WG reflected interdisciplinary expertise, including researchers, clinicians, and epidemiologists with specific knowledge of sarcopenia and its broader implications. The GLIS launched a call to candidates, and applications regarding the outcomes of sarcopenia were received by email and reviewed by the three leaders of the WG. Members were selected based on their contributions to the field, ensuring representation from key opinion leaders, genders, and diverse geographic regions.

### Evidence synthesis and analytical approach

Each sub-WG followed a structured approach to synthesize evidence and provide recommendations. For any of the investigated outcomes, experts were instructed to prepare an approximately two-page summary of existing and published evidence, relying on systematic reviews, meta-analyses, and relevant cohort studies. Depending on the outcome investigated (e.g. dichotomous outcome or non-dichotomous outcome) and the evidence available, experts were proposed to include different key aspects in their report: the association between sarcopenia and the investigated outcome based on current evidence; the preferred tools for measuring the outcome; the cutoffs defining normal and abnormal values, and the minimum clinically important difference (MCID). When possible, experts were also asked to comment on the relevance of these outcomes in clinical practice. All operational and consensual definitions of sarcopenia were considered acceptable. The impact of sarcopenia’s specific intervention studies on the outcomes was not considered in the present review. Experts were encouraged to differentiate findings from studies that used physical performance as part of the sarcopenia definition from those that relied only on muscle mass and strength.

### Output and reporting

Each sub-WG produced an approximately two-page report summarizing the current state of evidence related to their assigned topic, key challenges, and knowledge gaps. These reports were synthesized into this article to provide a comprehensive overview of sarcopenia’s influence across multiple domains. Level of evidence from meta-analyses, longitudinal studies and cross sectional studies for each outcome was further categorized as high level of evidence, moderate level of evidence or low level of evidence based on the following criteria: strength of evidence, amount of evidence, precision of results, and consistency of results.

## Results

### Physical performance and mobility limitations

Physical performance is an objective measure of whole-body function related to mobility, encompassing multiple systems such as musculoskeletal, neurological, pulmonary, and cardiovascular. It reflects not only the ability to perform mobility-related tasks but also the speed and efficiency of performance under standardized conditions. Conversely, mobility limitation refers to the inability to perform such tasks at a specific rate or level of performance [21].

A large number of measurement tools to assess physical performance have been validated with some of them being further recommended by consensual experts statements [21]: i.e. the use of gait speed tests (including both short and long distances, and the Timed Up and Go (TUG) test), the Sit-to-Stand (STS) test, as well as the short physical performance battery (SPPB) test, which incorporates the assessment of balance, gait speed, and STS performance.

#### Evidence from systematic reviews and meta-analyses

Despite the recognized importance of physical performance as a predictor of health outcomes in older adults, the expert group did not identify systematic reviews or meta-analyses which have specifically examined the association between sarcopenia (defined by low muscle mass and strength) and physical performance or mobility limitations. Many available studies include physical performance metrics (e.g., gait speed) within the definition of sarcopenia, complicating the analysis of their relationship as separate outcomes.

#### Evidence from original studies and expert opinion

Several cross-sectional and longitudinal studies have investigated on the association between sarcopenia and physical performance/mobility, but the results remain inconclusive. As example, the cross-sectional data from the Toledo Study for Healthy Aging (TSHA) [22] showed significant associations between probable (defined by low muscle strength) and confirmed (defined by low muscle strength and mass) sarcopenia (EWGWOP2’s criteria) and reduced habitual gait speed in older men (odds ratio [OR] of 2.1, 95% CI 1.3–3.3 and 2.5, 95% CI 1.2–4.9), both p ≤ 0.010), while associations were weaker and non-significant in older women (OR of 1.4, 95% CI 0.9–2.2 and 2.2, 95% CI 0.9–5.6, both p = 0.084). Additionally, the English Longitudinal Study of Ageing (ELSA) [23] revealed that probable and confirmed sarcopenia (EWGSOP2’s criteria) predicted slow gait speed in both men and women at baseline. However, over eight years of follow-up, results highlighted that probable sarcopenia, but not confirmed sarcopenia, was associated with gait speed decline in women, but not in men.

Other studies have examined the individual association of sarcopenia components with mobility limitation. A pooled analysis including cross sectional data from eight cohorts found that low appendicular skeletal muscle mass index and weak handgrip strength were consistently associated with habitual gait speed below 0.8 m/s in both men and women. Similarly, STS performance was significantly related to skeletal muscle mass index and handgrip strength in older adults, indicating the relevance of these sarcopenia components to mobility limitations [24, 25].

A summary of evidence-based preferred tools, cut-off points and minimum clinically important differences (MCID) for physical performance and mobility limitations is available in Table 1.

**Table 1:** Summary of evidence-based preferred tools, cut-off points and minimum clinically important differences

Outcome	Test	Cut-off points	MCID
Physical performance and mobility limitations	4- to 10-m habitual gait speed test (m/s)	Limitations: < 0.6 m/s [76] < 0.8 m/s [77] < 1.0 m/s [78]	0.1 m/s [79, 80]
	5-rep sit-to-stand (s)	Limitations: ≥ 12 s [81, 82]	2.3 s [83]
	30-s sit-to-stand (number of repetitions)	Limitations: ≤ 12 repetitions [84]	2 repetitions [85]
	Short physical performance battery (0–12 score)	No limitations: 10–12 points Mild limitations: 8–9 points Moderate limitations.: 6–7 points Severe limitations:< 6 points [86−87]	1 point [89]
Disability in ADL	Katz index (0–6 score)	Independence.: 6 points Moderate dep.: 3–5 points Severe dep.: 0–2 points [90]	0.5 points [91]
	Barthel index (0–100 score)	Independence: 100 points Slight dependence: 91–99 points Moderate dependence: 61–90 points Severe dependence: 21–60 points Total dependence: 0–20 points [92]	20 points [93]
Disability in IADL	Lawton index (0–8 score)	Slight to severe dependence: < 8 points in women < 5 points in men* [94]	0.5 points [91]
Quality of life	SarQoL questionnaire	No cut-off available to define low, medium or high HRQoL	Smallest Detectable change of 7.35 [95]. MCID NA

*The cut-off point is different between women and men to avoid potential gender bias

*ADL* basic activities of daily living, *IADL* instrumental activities of daily living, *MCID* minimum clinically important difference, *NA* not available

### Disability in basic and instrumental activities of daily living (ADL and IADL)

Instrumental and basic activities of daily living (IADL and ADL, respectively) are self-reported measures of function and ability that generally indicate a person’s capacity in the social and physical context in which functioning actually takes place [26]. The decline in IADL and ADL functioning has been shown to be a strong predictor of higher comorbidity and mortality in older people. Therefore, promoting and maintaining older individuals' functional status in IADL and ADL is considered one of the key principles of geriatric care [27].

Different tools exist to measure IADL and ADL, often in the form of questionnaires. So far, the Lawton index is considered the preferred tool to measure IADL due to the largest supporting evidence, which allows to make an informed decision in selecting the measurement tool [28]. For ADL, four instruments are considered to be the most adequate: the Functional Autonomy Measurement System (SMAF) [29], the 5-items Katz list (although content and wording were found to be often inconsistent across studies), the Functional Independence and Difficulty Scale (FIDS), and the Barthel Index [30].

#### Evidence from meta-analyses

The expert group did not identify systematic reviews or meta-analyses which specifically examined the association between sarcopenia and disability in IADL or ADL. However, indirect insights come from a meta-analysis published by Wang et al. in 2020 [31], revealing that, separately, reduced muscle mass, strength, and physical performance predict IADL and ADL declines over time. These findings underscore that the core components of sarcopenia significantly contribute to functional impairments in older adults.

#### Evidence from original studies and expert opinion

One prospective cohort study published by Da Silva Alexandre et al. in 2014 [32] reported that sarcopenia (EWGSOP1’s criteria) increased the risk of incident IADL disability by 2.3-fold during four years of follow-up. However, no consistent association with ADL was observed.

Additional evidence assessing the link between sarcopenia and IADL and ADL came mainly from cross-sectional studies. Among other examples is the Toledo Study for Healthy Aging that reported a 4.3-fold higher likelihood of IADL limitations in older women with sarcopenia (EWGSOP2’s criteria) compared to women without sarcopenia, non-consistent in men and not consistent for ADL, once again [22]. The SarcoPhAge study [33], a Belgian cohort study including 535 community-dwelling individuals, highlighted that women with sarcopenia (EWGSOP1’s criteria) were more dependent on specific IADL tasks, such as handling finances and housekeeping but did not show differences in total IADL or ADL scores. The Hisayama Study [34], on the other hand, showed a 2.5-fold increase in the probability of ADL disability, defined by a Barthel index score below 95, among individuals with sarcopenia (AWGS’s criteria) compared to their non-sarcopenic counterparts. Finally, in another cross-sectional study involving a community-dwelling oldest old sample, the probability of disability in ADL (≥ 1 ADL limitation) was two-fold higher in those with sarcopenia (AWGS’s criteria) compared to the participants without sarcopenia [35]. In institutionalized populations, sarcopenia (EWGSOP2’s criteria) was associated with lower ADL scores, although no relationship with IADL was observed [36]. These findings align with the notion that functional decline often follows a hierarchical pattern, with IADL limitations preceding ADL impairments. IADLs are cognitively more demanding than ADLs [28]. Therefore, the ability to perform IADL is frequently lost first, followed by the decline in mobility, and finally, the deterioration and loss of ADL.

### Quality of life

#### Evidence from systematic reviews and meta-analyses

Due to the negative influence of sarcopenia on clinical adverse outcomes, it is expected that sarcopenia may also negatively influence quality of life (QoL). The earliest systematic review published on this topic was conducted by Woo et al. in 2016 [37], and explored the relationship between sarcopenia components (e.g., muscle mass, strength, and physical performance) and health-related QoL (HRQoL). The findings of this review suggested that muscle strength and physical performance, rather than muscle mass alone, were more closely associated with HRQoL. Importantly, the unique study that used a comprehensive sarcopenia definition (i.e., EWGSOP1) demonstrated associations between sarcopenia and lower scores on the general health and physical functioning domains of the Short Form-36 (SF-36). The authors highlighted the poor uptake of consensus diagnostic criteria for sarcopenia. In light of this statement, Beaudart et al. [38] published another systematic review in 2023, synthesizing data from 43 published observational studies using specifically a consensual diagnostic criterion to characterize sarcopenia. This meta-analysis revealed that individuals with sarcopenia had significantly lower QoL scores than individuals without sarcopenia (Standardized Mean Difference (SMD): −0.76, 95% CI −0.95 to −0.57). Among the included studies, 19 used the EWGSOP1’s criteria, 15 used the EWGSOP2’s criteria, 9 used the AWGS criteria, and 2 used the FNIH criteria. The lowest difference in QOL between individuals with sarcopenia and those without was observed with the EWGSOP1’s criteria (23.826 individuals) and the largest difference with the AWGS criteria (1239 individuals).

Additionally, when using the SarQoL, a sarcopenia-specific quality of life questionnaire, greater differences of HRQoL values were found between individuals with sarcopenia and those without (SMD: −1.09) compared to the use of generic tools like SF-36 or EQ-5D. Therefore, Beaudart et al. published in 2024 an individual patient data meta-analysis with a focus on the specific HRQoL questionnaire, the SarQoL [11]. This analysis, including 32 databases with observational data from 5,116 participants, confirmed that individuals with sarcopenia experienced significant QoL reductions compared to those without sarcopenia (MD −12.32, 95% CI −15.27 to −9.37). Subgroup analyses confirmed that AWGS diagnostic criteria were associated with the most considerable QoL differences (MD: −17.65).

#### Evidence from original studies and expert opinion

Cross-sectional studies have consistently highlighted the negative association between sarcopenia and reduced HRQoL across diverse populations and settings [39]. Notably, the association between sarcopenia and a reduced quality of life was stronger in nursing home residents compared to community-dwelling older adults [38].

Over the past two decades, there has been a notable shift in health systems towards a more patient-centered model of care. This transition has been driven by various stakeholders, including clinicians, pharmaceutical industries, and regulatory agencies, all of whom have come to recognize the importance of integrating patient-reported outcomes (PROs) alongside traditional biomarkers of health improvement [40, 41]. This recognition has underscored the significance of considering not only clinical indicators but also the subjective experiences and perspectives of patients. Given that QoL measures have been shown to be significant predictors of hard clinical outcomes, such as hospitalization or mortality, their assessment is of crucial importance in clinical practice and observational and interventional research.

### Falls and fractures

#### Evidence from systematic reviews and meta-analyses

Sarcopenia is a well-documented risk factor for both falls and fractures, as evidenced by multiple systematic reviews and meta-analyses. Regarding falls, a meta-analysis by Beaudart et al. in 2017 [10] identified two prospective studies in community-dwelling older adults reporting that sarcopenia, diagnosed with EWGSOP1’s criteria, significantly increased the likelihood of recurrent falls. In 2020, Zhang et al. [42] analyzed data from 10 cohort studies, including 10,073 participants, confirming increased odds for falls in individuals with sarcopenia (OR: 1.52, 95% CI: 1.32–1.77). Results remained comparable regardless of sarcopenia definition adopted. However, this relationship was only observed in community-dwelling individuals and not in nursing home residents, suggesting context-specific differences. An umbrella review published the same year, encompassing 30 meta-analyses, reinforced these findings, reporting a relative risk (RR) of 1.75 (95% CI 1.55–1.97) for falls among community-dwelling older adults [43].

Regarding fractures, a systematic review published by Su et al. in 2017 [44] found that sarcopenia, assessed using EWGSOP1’s criteria, was associated with increased incident fracture rates (HR: 9.66, 95% CI 5.07–18.38) in community-dwelling older adults. In 2018, another meta-analysis evaluated data from nine cohort studies with 25,648 participants and reported a pooled RR of 1.34 (95% CI 1.13–1.58) [45]. Subgroup analyses identified stronger associations for men and studies using AWGS criteria. In 2021, the higher risk among men with sarcopenia was confirmed, whatever the sarcopenia criteria used for the definition, in a meta-analysis involving more than 7000 men from the United States, Sweden and Hong Kong with hazard ratios [HR] (adjusted to prior falls, BMD and fracture risk assessment tool (FRAX) probability) ranging from 1.39 to 2.07 [46]. Finally, in 2022, another meta-analysis of five cohort studies (27,990 participants) [47] confirmed sarcopenia’s association with fractures (adjusted HR: 1.50, 95% CI 1.08–2.08), particularly among older adults and those with hip fractures.

#### Evidence from original studies and expert opinion

To date, most studies considering sarcopenia and clinical outcomes have typically been undertaken in developed countries, likely due to the specialized equipment such as DXA devices (or BIA) required to assess muscle mass or its related compartments. In recognition of this, Veronese et al. in 2021 [48] examined, using a cross-sectional design, 13,101 community-dwelling older adults in five low- and middle-income countries, where sarcopenia criteria included low skeletal muscle mass, derived from age, sex and race-specific prediction equations, in the presence of weak grip strength or slow gait speed. The study found that individuals with sarcopenia had a higher prevalence of fall-related injuries (7.9% vs. 4.3% in individuals without sarcopenia) and greater odds of such injuries (OR: 1.85, 95% CI 1.24–2.77). In contrast, a prospective study showed no association between sarcopenia and the incidence of falls and fractures, however, low grip strength by itself was associated [49].

Reduced muscle strength, impaired balance, and diminished physical function are the mechanisms linking sarcopenia to falls. Collectively, these mechanisms increase vulnerability to environmental hazards and recovery challenges [50, 51]. Similarly, fractures are primarily caused by falls, with sarcopenia acting as an independent risk factor through reduced bone mineral density (BMD) and weakened muscle function. Evidence suggests muscle strength is more strongly associated with fracture risk, while muscle mass correlates with low bone mass [50].

### Hospitalization

#### Evidence from systematic reviews and meta-analyses

The expert group did not identify systematic reviews or meta-analyses that specifically examined the association between sarcopenia and the risk of hospitalization. However, numerous observational studies have been published in scientific literature reporting either the prevalence of sarcopenia among hospitalized individuals or examining the risk of hospitalization among individuals diagnosed with sarcopenia. Therefore, the group decided to perform a rapid review to simplify the summary of the available evidence about the link between sarcopenia and hospitalization.

#### Evidence from original studies and expert opinion

Sarcopenia is highly prevalent in hospitalized populations, with prevalence estimates varying significantly based on diagnostic criteria, population characteristics, and hospital settings. The meta-analysis performed by the group (unpublished), including 48 studies prevalence studies, found a pooled prevalence of 35% in hospitalized patients (95% CI 0.35–0.36, I^2^ = 98%), with rates ranging from 6 to 80%. For studies where only sex-specific prevalences were reported (3 in total), the prevalences ranged from 7.7%, 19.4%, and 40% in men and 4.7%, 15% and 23.1% in women. Many studies did not report the prevalence of severe sarcopenia, but when available, prevalences were as high as ~ 53% for men and 72% for women. Diagnostic criteria significantly influenced these estimates: prevalence was lowest with FNIH criteria (12%, 95% CI 0.09–0.15) and highest with AWGS criteria (57%, 95% CI 0.56–0.58). Prevalence also varied by setting, being higher in post-acute rehabilitation wards (47%, 95% CI 0.45–0.49) compared to acute geriatric wards (29%, 95% CI 0.28–0.30).

The rapid review has also aimed to report the potential increased risk of hospitalization associated with sarcopenia. No less than 15 prospective longitudinal studies published between 2013 [52] and 2024 [53] were identified and reported the risk of hospitalization during follow-up periods ranging from 3 months to 7 years, involving between 97 [54] and 4,000 individuals [55]. Approximately half of the studies used the EWGSOP2’s criteria to diagnose sarcopenia, while 2 used the EWGSOP1’s criteria, 5 used the FNIH criteria, and 1 used the AWGS criteria. The association between sarcopenia and an increased risk of hospitalization was mixed: half of the studies reported no significant association, while the other half reported a significantly higher risk of hospitalization for individuals with sarcopenia. Pooled HR ranged from 1.53 [56] to 2.25 [57] in studies adjusting for confounders such as age, sex, comorbidities, and cognitive impairment.

### Nursing home admissions

#### Evidence from systematic reviews and meta-analyses

The expert group did not identify systematic reviews or meta-analyses that specifically examined the association between sarcopenia and nursing home admission. Only a systematic review exploring the association between geriatric syndromes and nursing home admissions has been published by Wang et al. in 2013 [58]. While it did not focus on sarcopenia specifically, the review demonstrated that geriatric syndromes independently increase the risk of nursing home placement.

#### Evidence from original studies and expert opinion

In a longitudinal study published in 2022, Pacifico et al. [59] showed that sarcopenia, assessed in geriatric rehabilitation inpatients, predisposed individuals to a higher incidence of institutionalization within three months of discharge. Using different diagnostic criteria, the prevalence of sarcopenia in this cohort was 37.9% (EWGSOP1), 18.6% (EWGSOP2), and 26.1% (AWGS).

Cross-sectional studies provide additional context by documenting the high prevalence of sarcopenia among nursing home residents. Escribà-Salvans et al. [60] reported in 2022 a sarcopenia prevalence rates consistent with EWGSOP2’s criteria among older adults in nursing homes, highlighting its significant burden in institutionalized populations. Velázquez-Alva et al. [61] in 2020 demonstrated an association between sarcopenia, poor nutritional status, and chronic conditions such as type 2 diabetes in Mexican women living in nursing homes. Although direct evidence linking sarcopenia to incident nursing home admissions is sparse, outcomes commonly associated with sarcopenia, such as loss of self-care ability, falls, and fractures, are well-documented contributors to institutionalization. This indirect relationship underscores the role of sarcopenia in diminishing functional independence and necessitating nursing home placement.

### Mortality

#### Evidence from systematic reviews and meta-analyses

Sarcopenia is a significant predictor of mortality, as demonstrated by numerous systematic reviews and meta-analyses [10, 62–65]. A meta-analysis from 2016 [63], which included 10 studies with 3,797 participants, found that sarcopenia (EWGSOP1’s criteria) nearly doubled the mortality risk (HR: 1.87, 95% CI 1.61–2.18). The prevalence of sarcopenia in these studies varied widely, ranging from 10% to 41.2%, depending on the diagnostic method used. Another meta-analysis conducted in 2017 [64] analyzed six studies involving 7367 participants aged 70–84 years and reported a pooled HR of 1.60 (95% CI 1.24–2.06), indicating a 60% increase in mortality risk for individuals with sarcopenia. Subgroup analyses from this review showed that shorter follow-up periods (< 5 years) were associated with even higher risks (HR: 2.09, 95% CI 1.21–3.60), suggesting that sarcopenia’s effects on survival may be acute as well as chronic.

The most recent evidence are provided by a 2022 meta-analysis [62] synthesizing data from 56 studies with over 42,000 participants across diverse settings, including community-dwelling adults, hospitalized patients, and nursing home residents. The follow-up period ranged from 31 to 180 months for community-dwelling adults, 12–108 months for outpatients, 3–84 months for inpatients, and 6–24 months for nursing home residents. This analysis reported a pooled HR of 2.00 (95% CI 1.71–2.34) (40 longitudinal studies included in the model) and an OR of 2.35 (95% CI 1.64–3.37) (16 longitudinal studies included in the model), underscoring sarcopenia’s role in mortality irrespective of diagnostic criteria or population. In nursing home residents, the evidence was particularly compelling with a pooled HR obtained by two studies of 2.84 (95% CI 1.40–5.73).Studies that utilized bioelectrical impedance analysis (BIA) to estimate muscle mass or which had longer follow-up durations tended to report stronger associations, emphasizing the variability introduced by different methodologies.

#### Evidence from original studies and expert opinion

Recent studies using the updated EWGSOP2’s criteria have added further granularity to our understanding of sarcopenia’s effect on mortality [66–68]. Severe sarcopenia, particularly when associated with reduced gait speed (< 0.8 m/s), emerged as the most consistent predictor of mortality across multiple populations. In geriatric rehabilitation inpatients, the combination of low muscle mass and strength was linked to increased short-term (3-month) and long-term (1-year) post-discharge mortality [69]. Interestingly, studies consistently demonstrated a strong association between muscle strength and mortality, with muscle strength serving as a reliable predictor of increased mortality risk [70]. In contrast, other components of sarcopenia, such as muscle mass alone, demonstrated less consistent findings, with the literature reporting more heterogeneous results [71, 72].

These findings align with expert opinions that attribute sarcopenia’s mortality risks to its systemic effects, and suggested interrelationships between muscle mass and function [73]. Mechanistically, sarcopenia exacerbates metabolic dysregulation, chronic inflammation, and frailty, which in turn heighten susceptibility to adverse health outcomes like infections, cardiovascular events, and loss of mobility, ultimately contributing to higher mortality rates [74, 75]. However, a single common risk factor may explain the associations between sarcopenia and mortality, such as weight loss, inflammation, lack of physical activity etc.

## Discussion and conclusion

**Fig. 1:**
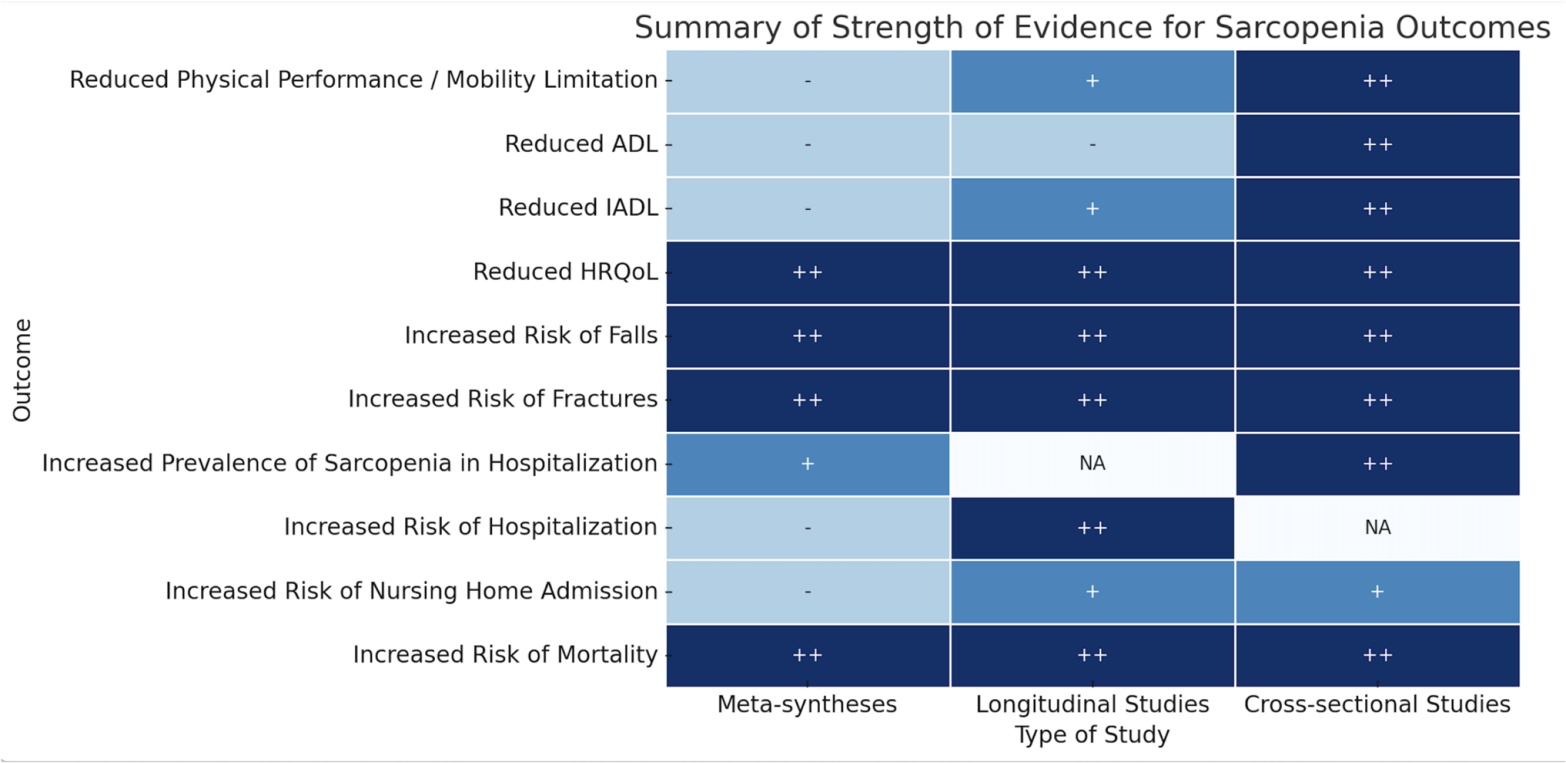
Strength of evidence for direct association found between sarcopenia across different outcome measures. A heatmap representation of evidence levels for various outcomes, categorized by high (++), moderate (+), and poor (−) evidence; *NA* non applicable, *AD*L basic activities of daily living, *IADL* instrumental activities of daily living

The systematic reviews, meta-analyses, and observational studies reviewed by the GLIS WG on outcome of sarcopenia and its seven subWGs revealed that sarcopenia is consistently and strongly associated with several hard clinical outcomes. The following associations have been considered with a high level of evidence from meta-syntheses, longitudinal studies and cross-sectional studies (Fig. 1):

- **Increased risk of mortality**: Numerous meta-syntheses have revealed that sarcopenia nearly doubled the risk of mortality, independently of the criteria used for the diagnosis of sarcopenia. Looking at components of sarcopenia separately, reduced muscle strength seems to be the highest predictor of death, whereas reduced muscle mass alone shows less consistent findings.
- **Reduced HRQoL**: Sarcopenia’s negative influence on HRQoL has been consistently reported in scientific literature, particularly in nursing home residents and when using sarcopenia-specific tools. A large meta-analysis including 43 cross-sectional studies has revealed that individuals with sarcopenia had significantly lower QoL scores than those without the condition, with the lowest difference found when using the EWGSOP1’s criteria and the largest difference found when applying the AWGS criteria. Looking at components of sarcopenia separately, another systematic review has reported that muscle strength and physical performance, rather than muscle mass alone, were more closely associated with HRQoL.
- **Increased risk of falls and fractures**: Numerous meta-syntheses have reported this association independently of the criteria used to diagnose sarcopenia. A higher risk of falls was mainly observed in community-dwelling individuals and not in nursing home residents, suggesting context-specific differences. Looking at components of sarcopenia separately, evidence suggests that muscle strength is more strongly associated with fracture risk, while muscle mass correlates with low bone mass.

For the other outcomes, the level of evidence varied depending on the type of study found.

A reduced IADL associated with sarcopenia has been consistently found in observational cross-sectional studies and longitudinal studies. One exception, however, is observed in nursing home residents. The majority of the available data have been obtained for a population with sarcopenia diagnosed with EWGSOP1’s criteria. Looking at components of sarcopenia separately, a meta-analysis has highlighted that reduced muscle mass, strength, and physical performance all predict IADL declines over time. Evidence linking sarcopenia to a reduction of ADL is however inconsistent so far, with one longitudinal study and two cross-sectional studies reporting no association, and a further three cross-sectional studies reporting a significant association between sarcopenia and reduced ADL.

Currently, restricted and inconsistent evidence has been reported on the association between sarcopenia and reduced mobility or physical performance. This is likely to be a result of physical performance measures being incorporated into the previous definition of sarcopenia, such that few studies would also include physical performance as outcomes. The level of evidence from meta-syntheses has been considered as low, from longitudinal studies as moderate and from cross-sectional studies as high.

For hospitalization risk, while studies are consistent with the fact that a high prevalence of sarcopenia is observed in hospitalized older adults, the fact that sarcopenia may be a predictor of hospitalization is less clear. Indeed, approximately half of the studies reported no significant association, while the other half reported a significantly higher risk of hospitalization for individuals with sarcopenia.

Evidence linking sarcopenia to incident nursing home admissions is sparse as well. Low level of evidence has been found from meta-synthesis and only moderate of evidence has been found from longitudinal and cross-sectional studies.

### Current gaps in evidence and perspectives

Despite significant advancements in sarcopenia research, several critical gaps remain. One of the major limitations is the scarcity of longitudinal studies reporting the medium-to-long term influence of sarcopenia on the different outcomes. These longitudinal studies are important to provide valuable insights into the temporal relationship between sarcopenia and key outcomes. Another prominent issue is diagnostic heterogeneity, including inconsistencies in sarcopenia definitions, measurement tools, and applied cut-off values. This variability limits comparability across studies but also robustness of the conclusions regarding sarcopenia’s association with key outcomes. In particular, because poor physical functioning is a well-established risk factor for most of the outcomes examined in this manuscript, when physical functioning is included as a diagnostic criterion for sarcopenia, there is an artificial inflate of the observed association between sarcopenia and these outcomes. As the GLIS criteria will include only low muscle and low muscle strength to be part of the definition, further research—preferably longitudinal studies with a sufficient length of follow-up—is needed to explore the relationship between sarcopenia, as defined by GLIS, and these outcomes.

Another challenge in the field is the difficulty in disentangling the independent and combined roles of muscle mass, muscle strength, and physical performance in relation to these outcomes. While sufficient evidence suggests that muscle strength is the primary driver of the association between sarcopenia and mortality, as it has been consistently linked to increased mortality risk, the relative contributions of muscle mass and physical performance remain unclear and require further investigation. For other outcomes, such as disability, quality of life, and falls, it is still uncertain whether muscle strength or muscle mass plays a more prominent role in these associations. Therefore, new longitudinal studies using the GLIS criteria should also include assessments of muscle mass and muscle strength separately, allowing for a clearer understanding of their respective contributions to these outcomes.

Additionally, systematic reviews and meta-analyses synthesizing available evidence remain limited or absent in several areas, particularly for functional decline, disability (IADL/ADL), nursing home admissions, and hospitalization. The lack of such comprehensive analyses hampers the ability to derive robust conclusions and identify priority areas for intervention.

Geographical and population-specific gaps are also evident. Research remains disproportionately centered on high-income countries, with limited data from low- and middle-income regions, despite the global relevance of sarcopenia. Similarly, certain high-risk populations, such as individuals in oncology and palliative care settings, remain underrepresented in sarcopenia research, particularly concerning mortality outcomes. Also, the variability in access to hospital settings/home care, cultural caregiving practices, and the economic burden of institutional care complicate evaluations of sarcopenia’s role in nursing home and hospital admissions.

## Conclusion

Sarcopenia is a pervasive yet underrecognized condition that significantly impacts aging populations worldwide. The findings from the GLIS working groups demonstrate the condition’s broad implications, from increased mortality and diminished HRQoL to heightened risks of falls, fractures, hospitalization, and reduced IADL. Addressing sarcopenia requires a multi-faceted approach, integrating early detection, standardized diagnostics, tailored interventions, and public health initiatives. Future research must focus on bridging existing gaps, particularly through longitudinal studies, to enhance understanding and improve outcomes for individuals with sarcopenia. As life expectancy continues to rise globally, tackling sarcopenia will be essential to promote healthy aging and to reduce the burden of age-related diseases.

## Data Availability

No datasets were generated or analysed during the current study.
